# Inhibition of tick-borne encephalitis virus in cell cultures by ribavirin

**DOI:** 10.3389/fmicb.2023.1182798

**Published:** 2023-06-12

**Authors:** Wan-Da Tang, Hai-Lin Tang, Hao-Ran Peng, Rui-Wen Ren, Ping Zhao, Lan-Juan Zhao

**Affiliations:** ^1^Department of Microbiology, Faculty of Naval Medicine, Naval Medical University, Shanghai, China; ^2^Center for Disease Control and Prevention of Southern Theater Command, Guangzhou, China

**Keywords:** tick-borne encephalitis virus, ribavirin, myxovirus resistance A, signal transducer and activator of transcription 3, tumor necrosis factor alpha

## Abstract

Tick-borne encephalitis virus (TBEV) belonging to arboviruses is a major member of zoonotic pathogens. TBEV infection causes severe human encephalitis without specific antiviral drugs. Due to its use of antiviral drug against a wide range of viruses, we investigated antiviral effect of ribavirin against TBEV in susceptible human cell lines A549 and SH-SY5Y. Ribavirin displayed minor cytotoxicity on multiple cell lines. Ribavirin obviously impaired TBEV replication and protected the infected cells from cytopathic effect. Importantly, ribavirin markedly inhibited TBEV propagation, as evidenced by impairment of TBEV production and viral RNA replication. Treatment with ribavirin (co-treatment and post-treatment) led to a dose-dependent reduction in TBEV titers as well as the viral RNA levels. Antiviral protein myxovirus resistance A mRNA expression was significantly up-regulated and signal transducer and activator of transcription 3 was activated in TBEV-infected A549 cells upon the ribavirin treatment. Induction of inflammatory cytokine tumor necrosis factor alpha by TBEV was decreased in A549 cells with the treatment of ribavirin, whereas interleukin 1 beta release appeared to be unaffected. These results suggest that ribavirin might represent a promising safe and effective antiviral drug against TBEV.

## Introduction

Arboviruses comprise several significant human pathogens such as dengue virus, Zika virus, Japanese encephalitis virus, Crimean-Congo hemorrhagic fever virus, tick-borne encephalitis virus (TBEV), yellow fever virus, West Nile virus and Chikungunya virus. Arboviruses are responsible for considerable morbidity and mortality in vertebrates and humans. TBEV, a major member of arboviruses, is a positive-stranded RNA virus belonging to the *Flavivirus* genus in the Flaviviridae family. TBEV genome encodes a polyprotein that is processed into three structural proteins including capsid, precursor membrane and envelope glycoproteins and seven non-structural (NS) proteins including NS1, NS2A, NS2B, NS3, NS4A, NS4B and NS5 (Blom et al., 2018). In nature, TBEV is maintained in transmission cycles between Ixodes ticks and wild mammalian hosts. Although transmission of unpasteurized milk as well as milk products from infected stock was reported, TBEV, a zoonotic pathogen, is transmitted to humans primarily *via* bites of infected Ixodes ticks (Yoshii, 2019). As a tick-borne viral pathogen of humans, TBEV is becoming an international public health concern.

Infection with TBEV causes severe neurological manifestations including meningitis, encephalitis and meningoencephalitis with and without permanent sequelae and even results in death (Pulkkinen et al., 2018). The geographic distribution of TBEV is considered mainly in large areas of Europe and Asia (Im et al., 2020). In mainland China, TBEV is an endemic pathogen in the Northeast and the Xinjiang and the distribution is closely related to the distribution of tick vectors (Gao et al., 2010). There are five licensed vaccines to TBEV infection on the basis of formalin-inactivated and purified whole virus of TBEV strains. However, some reports have shown the vaccine failures in the particular individuals (Blom et al., 2018). There are no specific antiviral drugs available for TBEV infection (Yoshii, 2019). In recent years, the incidence of diseases caused by TBEV has increased.

Since discovery in 1972 as a synthetic nucleoside analog, ribavirin has been focused due to anticancer and antiviral biological properties. Ribavirin exerted clinical benefit in patients with acute myeloid leukemia and was shown as an anticancer therapy (Borden and Culjkovic-Kraljacic, 2010). The anticancer efficacy of ribavirin has been explored on preclinical models and clinical trials in acute myeloid leukemia, oropharyngeal squamous cell carcinoma, metastatic breast cancer and glioblastoma (Volpin et al., 2017; Casaos et al., 2019). The use of ribavirin as an anticancer agent is apparently instructive. Moreover, ribavirin is well known as a chemotherapeutic agent with activities against a wide range of RNA and DNA viruses. For instance, ribavirin in combination with pegylated interferon alpha (IFN-α) was established as the therapy for chronic hepatitis C (Reddy et al., 2009). Ribavirin therapy was also efficient for hepatitis E (De Winter et al., 2018). Ribavirin was clinically used to treat infection with respiratory syncytial virus as well as other noninfluenza respiratory viruses (Gross and Bryson, 2015; Tejada et al., 2022). Ribavirin has also been used to treat patients infected with Lassa fever virus or Crimean-Congo hemorrhagic fever virus (Ascioglu et al., 2011; Johnson et al., 2018; Cheng et al., 2022). In particular, ribavirin had antiviral efficacy in children with tick-borne encephalitis (Skripchenko et al., 2019). Although ribavirin is regarded as a safe antiviral drug, the effectiveness of ribavirin for the viruses remains to be validated and the precise mechanisms of ribavirin action are still not completely understood.

As no effective anti-TBEV treatments are available, it is necessary to develop antiviral drugs against TBEV for its expanding worldwide. Based on its antiviral activities and the antiviral efficacy in children with tick-borne encephalitis, we wondered whether ribavirin might play active roles in inhibiting TBEV propagation and eliciting antiviral response. In the present study, influence of ribavirin on TBEV propagation was evaluated in susceptible human cell lines by detecting viral protein, viral RNA, virus titer, antiviral protein and inflammatory cytokines. The underlying mechanisms by which ribavirin mediates antiviral and immunomodulatory effects were also discussed.

## Materials and methods

### Cell culture

Human lung adenocarcinoma A549 cells, human neuroblastoma SH-SY5Y cells, African green monkey kidney Vero cells and porcine kidney PK-15 cells were used in the study. All cell lines were grown in Dulbecco’s modified Eagle’s medium (DMEM) supplemented with 10% fetal bovine serum (FBS), 1% L-glutamine, 1% non-essential amino acids and 1% penicillin–streptomycin at 37°C under 5% CO_2_. These products for cell culture were from Invitrogen (United States). Vero cells were used to propagate TBEV. PK-15 cells were applied to determine virus titer. A549 and SH-SY5Y cells were chosen to evaluate anti-TBEV effects of ribavirin.

### Virus propagation

TBEV was stocked in the laboratory as described previously (Ding et al., 2022; Tang et al., 2022). TBEV was propagated in Vero cells, and cell culture supernatants collected at 72 h post-inoculation were centrifuged at 2,500 rpm for 10 min to remove cell debris. Aliquots of supernatant fraction were stored at −80°C until use. Virus titer was titrated on PK-15 cells by plaque assay. The experiments concerning TBEV infection were performed in Biological Safety Level 3 Laboratory in accordance with the guidelines by Committee on Safety of Biomedicine at Naval Medical University.

### Cell viability assay

Cells seeded in 96-well plates were cultured overnight to form a confluent monolayer. For 10 mg/mL stock, ribavirin (Millipore, United States) was dissolved in phosphate-buffered saline (PBS). The culture medium was removed and the cells were subsequently incubated with ribavirin at concentrations range of 0 to 300 μg/mL in fresh culture medium. After 48 h of incubation, cell viability was evaluated using One Solution Cell Proliferation Assay kit containing MTS according to the manufacture^’^s instructions (Promega, United States). The absorbance at 490 nm was read on a Synergy 2 Multi-Mode Microplate Reader (BioTek, United States).

### Immunofluorescence staining

A549 cells grown in 96-well plates overnight were inoculated with TBEV at a multiplicity of infection (MOI) of 0.1 and different concentrations of ribavirin in fresh culture medium. After 48 h, the medium was removed and the cells were fixed with 4% paraformaldehyde in PBS for 15 min at room temperature, washed twice with PBS, and permeabilized with methanol for 20 min at −20°C. After two washes with PBS and blocking with 3% bovine serum albumin (BSA) in PBS for 2 h, the cells were incubated with formaldehyde-inactivated TBEV immunized mouse ascites recognizing the viral surface antigen (1:500 dilution in 1% BSA) overnight at 4°C. The cells were washed three times with PBS and subsequently labeled with an anti-mouse goat secondary antibody conjugated with Alexa Fluor 488 (1:1,000 dilution in 1% BSA; Abcam, UK) in the dark for 1 h. Finally, the cells were treated with mounting medium with 4^′^,6^′^-diamidino-2-phenylindole (DAPI) (Abcam, UK) for 5 min to visualize the cell nuclei. The numbers of infected cells and total cells were counted by using Cytation 5 imaging reader (BioTek, United States), and the infection rate was calculated with Gen5 3.10 software. Images were acquired under a fluorescence inverted microscope (Olympus IX81, Japan).

### Ribavirin treatment

A549 or SH-SY5Y cells were seeded into 12-well plates and cultured overnight to form a confluent monolayer. To evaluate which stage of TBEV life cycle was affected by ribavirin, three schemes of ribavirin treatment were applied. For co-treatment, culture medium was removed and the cells were cultured for 48 h in fresh culture medium containing TBEV at an MOI of 0.1 and ribavirin at concentrations ranging from 0 to 50 μg/mL. For post-treatment, the cells were inoculated for 2 h with 0.1 MOI TBEV at 37°C. After removal of the viral inoculum, the cells were washed once with PBS, and grown in fresh culture medium containing ribavirin at the indicated concentrations for 48 h. For pre-treatment, the cells were incubated with ribavirin at the indicated concentrations in fresh culture medium for 12 h. After removal of the medium, the cells were washed once with PBS and inoculated for 2 h with 0.1 MOI TBEV at 37°C. Following the incubation, the viral inoculum was aspirated and replaced with fresh culture medium, and the cells were then cultured for 48 h. The time point was calculated from the end of the 2 h adsorption. As a solvent control, PBS was added to TBEV-infected cells at a final concentration of 0.1% (v/v). Culture supernatants, cellular RNA and cell lysates were collected for the following assays.

### Plaque assay

Confluent monolayer of PK-15 cells grown in 12-well plates was inoculated with 10-fold dilutions of the culture supernatants from TBEV-infected cells with and without the ribavirin treatment. After an incubation period of 3 h at 37°C, the viral inoculum was removed, and the cells were washed once with PBS. 2% carboxymethylcellulose (Sigma-Aldrich, United States) overlay medium was added to the cells. After 6 days, the overlay medium was removed, the cells were fixed with 4% paraformaldehyde for 15 min and stained with 1% crystal violet to visualize plaques. After 15 min of staining, the crystal violet was decanted and the plaques were counted. The virus titer was expressed as plaque-forming units (PFU)/mL.

### Enzyme-linked immunosorbent assay

The culture supernatants from A549 cells with and without the ribavirin treatment were collected for cytokine measurement. ELISA kits (R&D Systems, United States) were used to determine concentrations of human tumor necrosis factor alpha (TNF-α) and interleukin 1 beta (IL-1β) according to the manufacturer’s protocols. The data were acquired on the Synergy 2 Multi-Mode Microplate Reader (BioTek) and analyzed using SigmaPlot 10.0 software (Systat Software Inc.).

### Real-time reverse transcription PCR

Total cellular RNA was extracted with Trizol reagent (Invitrogen, United States). cDNA was generated from total RNA by using random hexamer primers and moloney murine leukemia virus reverse transcriptase kit (Promega). Quantitative real-time PCR was performed on cDNA templates using SYBR Green PCR kit (Promega). The signals were acquired on a Rotor-Gene 3,000 Thermal Cycler (Corbett, Australia), and ΔΔCt was calculated with Rotor-Gene 6.1.81 software. Glyceraldehyde-3-phosphate dehydrogenase (GAPDH) RNA levels were quantified as an endogenous reference for normalization of target genes. The primer sequences of target genes were used as follows: TBEV, 5^’^-TGGAYTTYAGACAGGAAYCAACACA-3^′^(forward) and 5^′^- TCCAGAGACTYTGRTCDGTGTGGA-3^′^(reverse); myxovirus resistance A (MxA), 5^’^-ACAGGACCATCGGAATCTTG-3^′^ (forward) and 5^′^- CCCTTCTTCAGGTGGAACAC-3^′^ (reverse); GAPDH, 5^’^-TGGGCTACACTGAGCACCAG-3^′^ (forward) and 5^’^-AAGTGGTCGTTGAGGGCAAT-3^′^ (reverse).

### Western blotting

Proteins in A549 cell lysates were electrophoretically separated on sodium dodecyl sulfate polyacrylamide gels and transferred onto polyvinylidene difluoride membranes. After being blocked with 5% nonfat dry milk in 0.1% Tween 20 in tris-buffered saline, the membranes were incubated with rabbit antibodies (1:1,000 dilution; Cell Signaling Technology, United States) for phospho-signal transducer and activator of transcription 3 (P-STAT3) (Tyr705) or GAPDH at 4°C overnight. The membranes were washed three times with 0.1% Tween 20 in tris-buffered saline and subsequently incubated with horseradish peroxidase conjugated goat anti-rabbit IgG (1:2,000 dilution) for 2 h at room temperature. The membranes were washed three times, and target proteins were visualized with enhanced chemiluminescent solution detection reagents (Bio-Rad, United States) on a GeneGnome HR image capture (Cambridge, UK).

### Statistical analysis

Data are shown as mean and standard deviation (SD). The statistical analysis was performed with two-tailed unpaired or paired Student’s *t*-test (GraphPad Prism 8.0) as indicated. Differences with *p* value <0.05 were considered statistically significant: *p < 0.05, **p < 0.005, ***p < 0.002, ****p < 0.001.

## Results

### Ribavirin has minor cytotoxicity on multiple cell lines

Some compounds were evaluated for their antiviral efficacy against TBEV on human lung adenocarcinoma A549 cells (Yu et al., 2013; Krol et al., 2019). Human neuroblastoma cell line SH-SY5Y was a valuable cell model for elucidating neuropathogenesis of neurotropic viruses (Yang et al., 2008; Laassri et al., 2011). As TBEV is a neurotropic virus, cell lines of extraneural as well as neuronal origin were chosen to be cell model for evaluating antiviral efficacy of ribavirin. In the present study, antiviral efficacy of ribavirin against TBEV was examined on A549 and SH-SY5Y cell lines.

**Figure 1:**
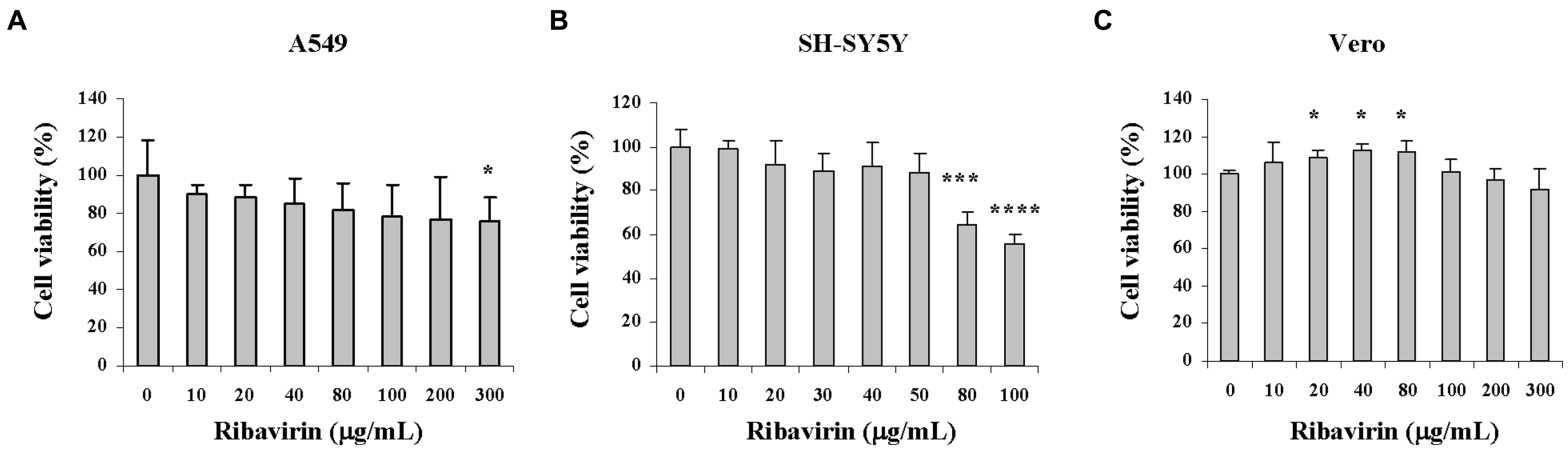
Cytotoxicity profile of ribavirin on multiple cell lines. Cells were cultured for 48 h in the presence of ribavirin at the indicated concentrations. Cell viability was determined by MTS assay. **(A)** A549 cell viability upon ribavirin treatment. **(B)** SH-SY5Y cell viability upon ribavirin treatment. **(C)** Vero cell viability upon ribavirin treatment. The results are representative of two independent experiments. Data are shown as mean ± SD (*n* = 3); *p < 0.05, ***p < 0.002, ****p < 0.001 compared with the untreated control (0 μg/mL).

We previously showed that 100 μg/mL of ribavirin displayed no observable toxicity on human hepatoma Huh7.5.1 cells (Zhao et al., 2012). To initially determine whether ribavirin had any effects on proliferation of the multiple cell lines used, A549, SH-SY5Y and Vero cells were treated for 48 h with ribavirin at the increasing doses (0–300 μg/mL). The cytotoxicity was determined in terms of cell viability by the MTS assay. As shown in Figure 1, ribavirin was differentially cytotoxic among cell lines and only high doses of ribavirin gave rise to cytotoxicity. In A549 cells, ribavirin did not exert significant cytotoxic effects at doses up to 200 μg/mL. Treatment with 300 μg/mL of ribavirin reduced A549 cell proliferation by 24% and exerted significant inhibitory effects compared with the untreated control (0 μg/mL, *p* < 0.05; Figure 1A). Instead, ribavirin at doses up to 50 μg/mL had little inhibitory effects on SH-SY5Y cell proliferation. The significant inhibitory effects on SH-SY5Y cell proliferation were detectable following the ribavirin treatment at doses of 80 and 100 μg/mL (*p* < 0.002, *p* < 0.001; Figure 1B). Ribavirin treatment was nontoxic to Vero cells even at a high dose of 300 μg/mL (Figure 1C). These results showed that cytotoxicity of ribavirin was associated with cell types and the minor cytotoxic effects may be favorable for its antiviral activity and clinical usage, suggesting a great safety profile of ribavirin. For evaluation of antiviral efficacy, the tested doses of ribavirin did not exceed 60 μg/mL with no cytotoxic effects on the cell lines tested.

### Ribavirin obviously impairs TBEV replication in A549 cells

**Figure 2:**
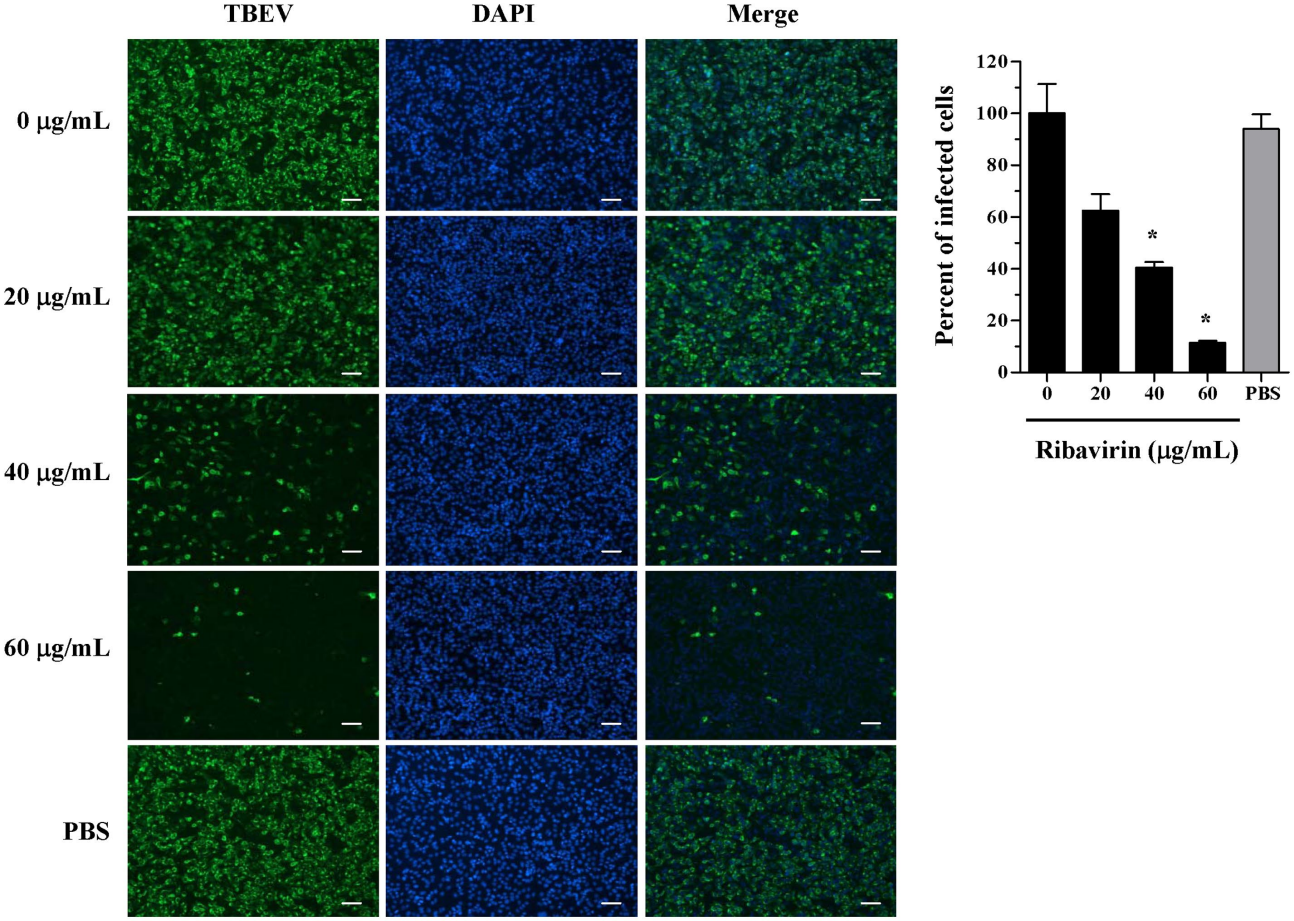
Inhibition of TBEV protein expression by ribavirin in A549 cells. A549 cells were incubated with TBEV and ribavirin at the concentrations ranging from 0 to 60 μg/mL or 0.1% PBS (solvent control). The cells were fixed at 48 h post-inocubation and stained with TBEV immunized mouse ascites and goat anti-mouse secondary antibody conjugated with Alexa Fluor 488 (green) and counterstained with DAPI (blue) by immunofluorescence staining. The representative images of three experiments are shown. Scale bar, 100 μm. The percentages of TBEV-infected A549 cells are shown as mean ± SD of three experiments (right panel). *p < 0.05 compared with the untreated control (0 μg/mL).

TBEV envelope protein expression was inhibited in the infected cells by some compounds (Eyer et al., 2016, 2017). We analyzed the influence of ribavirin on TBEV protein expression over the course of infection in A549 cells by immunofluorescence. A549 cells were incubated with TBEV and varying doses of ribavirin and the viral protein expression was measured at 48 h post-inocubation. As shown in Figure 2 (left panel), TBEV protein could be clearly detected in the infected cells without the ribavirin treatment (untreated control) and a high percentage of TBEV-positive cells was detectable (right panel), indicating that A549 cells are susceptible to TBEV. Treatment with PBS was used as a solvent control. As expected, PBS showed very minor influence on the viral protein expression. TBEV protein was undetectable in mock-infected A549 cells (data not shown). TBEV protein expression was persistently decreased in the infected cells with the ribavirin treatment compared to the untreated control. Ribavirin at the dose of 40 or 60 μg/mL showed a significant inhibition of the viral protein expression in TBEV-infected cells (*p* < 0.05; Figure 2, right panel). Treatment with 60 μg/mL of ribavirin led to nearly a complete inhibition of TBEV infection. Here immunofluorescence staining showed the dose-dependent inhibition of TBEV protein expression in the ribavirin-treated A549 cells. Moreover, morphological changes of TBEV-infected A549 cells upon ribavirin treatment were observed. In comparison with mock control, cytopathic effect was obviously detectable in the infected A549 cells, and such cytopathic effect was almost abolished due to the 50 μg/mL ribavirin treatment (data not shown). Ribavirin may protect the infected cells from the cytopathic effect by impairing TBEV replication.

### Ribavirin markedly inhibits TBEV propagation in A549 and SH-SY5Y cells

**Figure 3:**
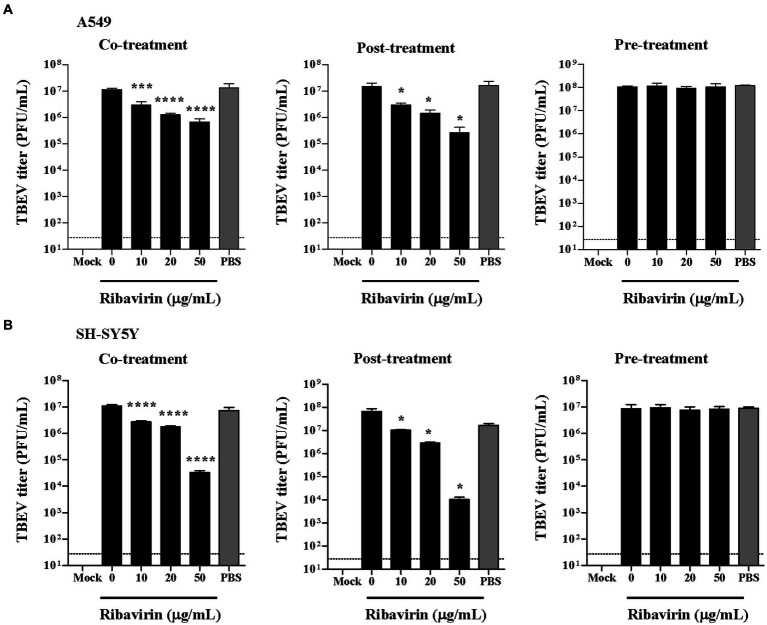
Effects of ribavirin on TBEV production in different cell lines. Cells were infected with TBEV and treated with the increasing concentrations of ribavirin at three schemes (co-treatment, post-treatment and pre-treatment). At 48 h post-inocubation, culture supernatants were collected and virus titers were monitored by plaque assay. **(A)** TBEV titers in A549 cells. **(B)** TBEV titers in SH-SY5Y cells. Data represent the mean ± SD of three experiments (*n* = 3); *p < 0.05, ***p < 0.002, ****p < 0.001 compared with the untreated control (0 μg/mL). Uninfected cells were used as a mock control (Mock). PBS was used as a solvent control. The horizontal dashed line indicates the minimum detectable threshold of 1.44 log_10_ PFU/mL.

Antiviral activity of polyphenol complex from seagrass of the Zosteraceae family against TBEV was analyzed based on several schemes of application of the compound (Krylova et al., 2018). To study the mechanisms of anti-TBEV activity of ribavirin, three schemes of ribavirin treatment were applied to A549 and SH-SY5Y cells. Both TBEV production and the viral RNA replication were examined in the infected cells with and without the ribavirin treatment. At 48 h post-inocubation, the culture supernatants and the cellular RNA were collected from the A549 and SH-SY5Y cells for plaque assay and real-time PCR analysis, respectively. Indeed, TBEV effectively propagated in the cell-based assay systems as evidenced that the peak levels of virus titer were 1.07 × 10^8^ PFU/mL in the culture supernatants from infected A549 cells and 6.67 × 10^7^ PFU/mL from the infected SH-SY5Y cells (Figure 3). For co-treatment, dose-dependent inhibition of TBEV production by ribavirin was observed in A549 and SH-SY5Y cells. TBEV titers from the ribavirin-treated cells were significantly reduced at doses of 10, 20 and 50 μg/mL compared with the untreated cells (0 μg/mL, *p* < 0.002, *p* < 0.001; Figures 3A,B, left panel). Similarly, dose-dependent inhibition of TBEV production by ribavirin post-treatment was also observable in A549 and SH-SY5Y cells. The post-treatment of ribavirin at doses of 10, 20 and 50 μg/mL significantly decreased the viral titers (*p* < 0.05; Figures 3A,B, middle panel). In comparison with the untreated cells, the highest tested dose of 50 μg/mL ribavirin led to a reduction in TBEV titer of about 100-fold in A549 cells and 1,000-fold in SH-SY5Y cells. By contrast, there were no antiviral effects of ribavirin pre-treatment observed in A549 and SH-SY5Y cells (Figures 3A,B, right panel). TBEV titers from the ribavirin pre-treated cells were almost the same as those from the untreated cells. As a solvent control, PBS treatment showed little or no effects on the viral titers. By using co-treatment as well as post-treatment scheme of application, the antiviral effects of ribavirin against TBEV were evident in A549 and SH-SY5Y cells. The two schemes of ribavirin treatment were thereby focused in the following assays.

**Figure 4:**
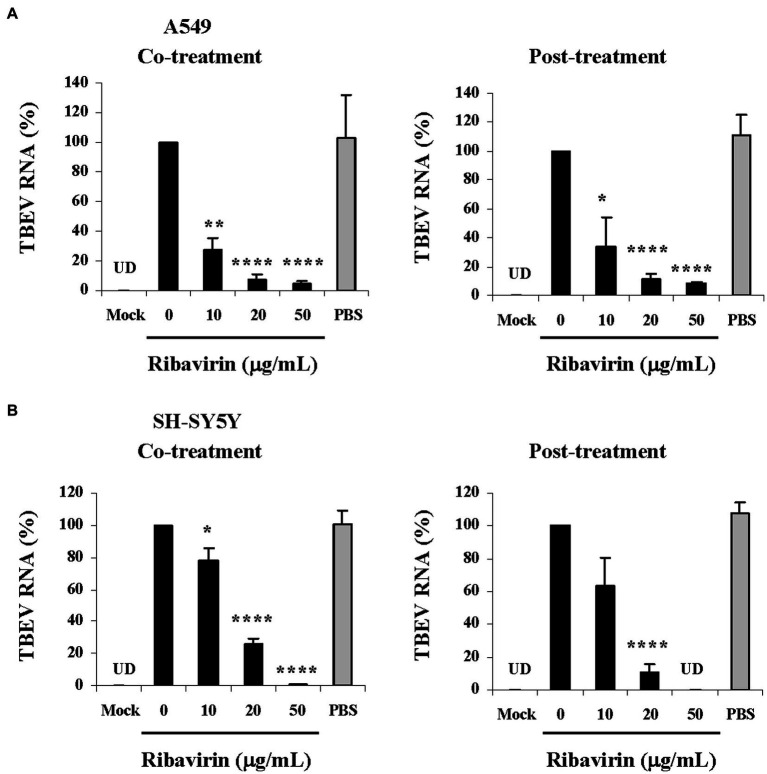
Effects of ribavirin on TBEV RNA replication in different cell lines. Cells were infected with TBEV and treated with the increasing concentrations of ribavirin at two schemes (co-treatment and post-treatment). At 48 h post-inocubation, total RNA extracts were prepared and levels of TBEV RNA were quantified by real-time reverse transcription PCR. **(A)** TBEV RNA levels in A549 cells. **(B)** TBEV RNA levels in SH-SY5Y cells. TBEV RNA levels are shown as relative percentages of the untreated control (0 μg/mL). For each sample, real-time PCR analysis was performed in triplicate. Data are from three experiments and represent the mean ± SD (*n* = 3); *p < 0.05, **p < 0.005, ****p < 0.001 compared with the untreated control (0 μg/mL). Uninfected cells were used as a mock control (Mock). PBS was used as a solvent control. UD, undetectable.

Next, the antiviral effects were further confirmed by measuring TBEV RNA levels in the cells with the co-treatment or post-treatment of ribavirin. In A549 cells, TBEV RNA levels were dose-dependently reduced by ribavirin at the co-treatment as well as the post-treatment scheme and significant differences (*p* < 0.05, *p* < 0.005, *p* < 0.001) were observed at all tested doses as compared with the untreated control (0 μg/mL; Figure 4A). In SH-SY5Y cells, the co-treatment or post-treatment of ribavirin also resulted in a dose-dependent reduction of TBEV RNA levels and such inhibitory effects were significant as compared with the untreated control (*p* < 0.05, *p* < 0.001; Figure 4B). Particularly, a complete inhibition of viral replication was observed at a dose of 50 μg/mL for ribavirin with the two treatment schemes in SH-SY5Y cells, which corresponded to the TBEV titer reduction of about 1,000-fold (Figure 3B, left and middle panel). Whereas there were no inhibitory effects observed on TBEV replication in the infected A549 and SH-SY5Y cells with PBS treatment (solvent control). Furthermore, the inhibition of TBEV replication by ribavirin was comparable with its reduction in the virus production based on the co-treatment as well as the post-treatment scheme. Together, ribavirin markedly inhibited TBEV propagation and exerted potent antiviral effects against TBEV in A549 and SH-SY5Y cells.

### Ribavirin up-regulates MxA gene expression and STAT3 phosphorylation in TBEV-infected A549 cells

**Figure 5:**
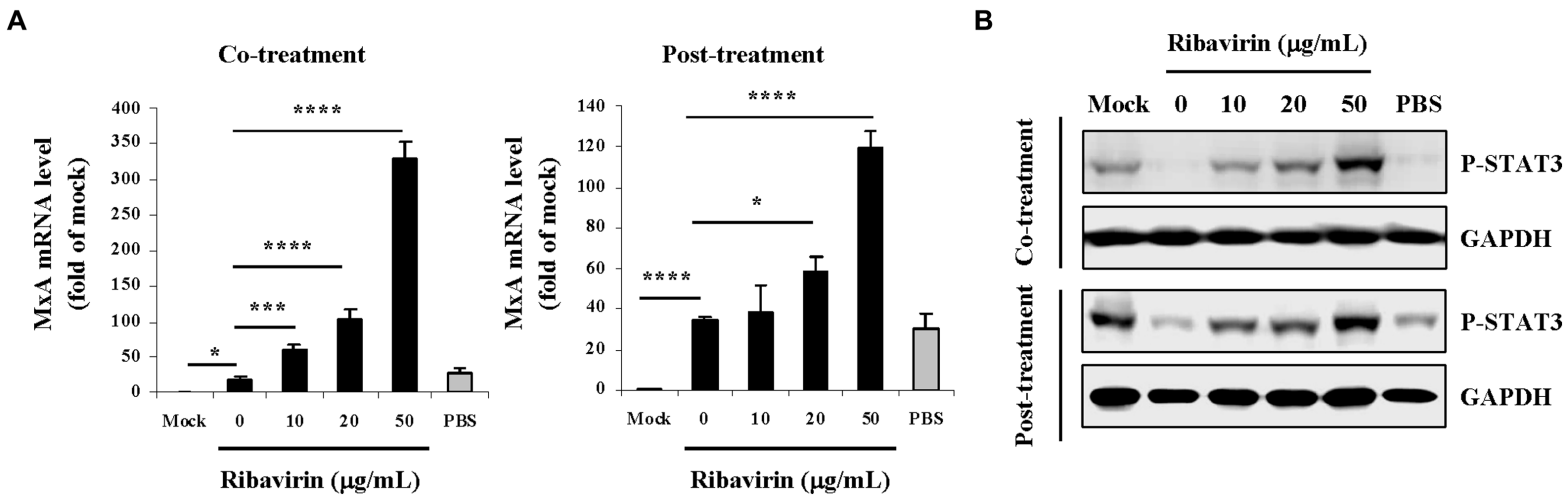
Effects of ribavirin on expression of MxA gene and phosphorylation of STAT3 in TBEV-infected A549 cells. Cells were infected with TBEV and treated with the increasing concentrations of ribavirin at two schemes (co-treatment and post-treatment). At 48 h post-inocubation, total RNA extracts and cell lysates were prepared. **(A)** MxA mRNA levels were quantified by real-time reverse transcription PCR. MxA mRNA levels are shown as fold of mock control. For each sample, real-time PCR analysis was performed in triplicate. Data are from three experiments and represent the mean ± SD (*n* = 3); *p < 0.05, ***p < 0.002, ****p < 0.001. **(B)** P-STAT3 was detected in the cell lysates by Western blotting. GAPDH is shown as a loading control. The results shown are representative of at least three independent experiments. Uninfected cells were used as a mock control (Mock). PBS was used as a solvent control.

The following experiments focused on antiviral and anti-inflammatory responses induced by ribavirin, which were investigated in TBEV-infected A549 cells. Since ribavirin exerted the inhibitory effects on TBEV propagation, which downstream elements mediated the antiviral response were then examined. As a member of IFN-stimulated genes whose protein products mediate a variety of specific antiviral response, MxA gene expression was analyzed in the TBEV-infected A549 cells with and without the ribavirin treatment. At 48 h post-inocubation, the RNA obtained from the cells upon the co-treatment or the post-treatment of ribavirin was also measured for MxA mRNA levels. Real-time PCR analysis showed that the MxA mRNA levels were significantly up-regulated in TBEV-infected A549 cells compared with the mock infected cells (Mock, *p* < 0.05 for the co-treatment, *p* < 0.001 for the post-treatment; Figure 5A), implying TBEV triggered antiviral response at the early stage of infection. The treatment of ribavirin at the co-treatment as well as the post-treatment scheme resulted in a dose-dependent increase in MxA mRNA levels and significant differences (*p* < 0.05, *p* < 0.002, *p* < 0.001) were detectable in the infected A549 cells treated with the ribavirin versus the untreated cells (0 μg/mL). The highest levels of MxA mRNA were detectable in the cells treated with 50 μg/mL of ribavirin. There were no significant differences in MxA mRNA levels in the infected A549 cells alone or along with PBS treatment (solvent control).

STAT3, a member of transcription factor family, is a key regulator of numerous physiological functions, including inflammation, cell proliferation, cell survival and cellular differentiation (Roca Suarez et al., 2018). STAT3 has been documented to play important roles in viral infection and pathogenesis (Chang et al., 2018). STAT3 is activated by tyrosine phosphorylation on Tyr705. At 48 h post-inocubation, the cell lysates obtained from A549 upon the co-treatment or the post-treatment of ribavirin was analyzed for STAT3 phosphorylation by Western blotting. As shown in Figure 5B, levels of phosphorylated STAT3 (P-STAT3) were noticeably impaired in the TBEV-infected cells (0 μg/mL) compared with those in the uninfected cells (Mock). Treatment with the ribavirin at the co-treatment as well as the post-treatment scheme led to a dose-dependent enhancement of STAT3 phosphorylation. PBS treatment had no enhanced effects on the STAT3 phosphorylation. Therefore, ribavirin induced activation of STAT3 during TBEV infection. These observations indicate that ribavirin may exert antiviral effects by up-regulating antiviral protein MxA gene expression and STAT3 phosphorylation in A549 cells infected with TBEV.

### Ribavirin differentially regulates inflammation cytokine production in TBEV-infected A549 cells

**Figure 6:**
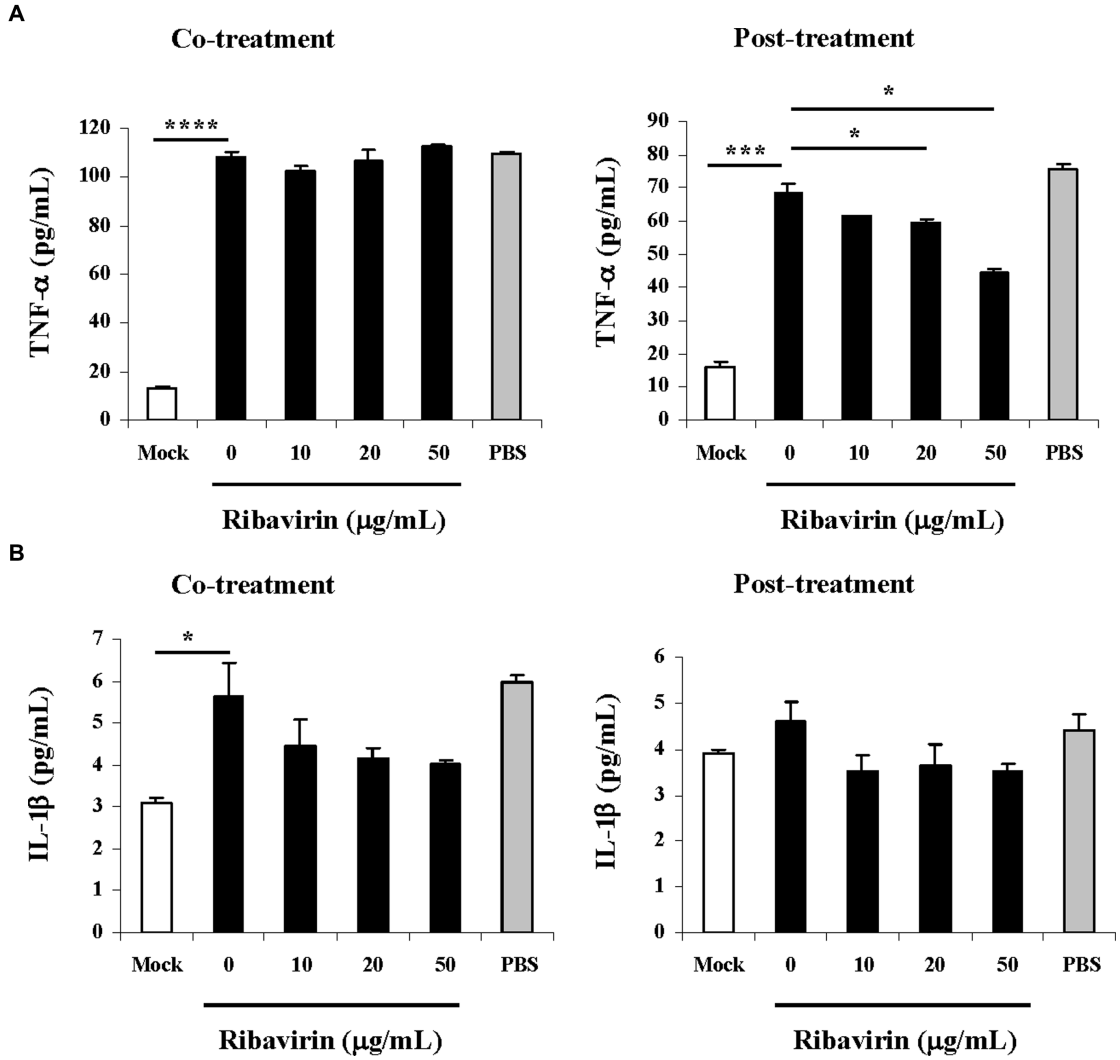
Cytokine response in TBEV-infected A549 cells upon ribavirin treatment. A549 cells were infected with TBEV and treated with the increasing concentrations of ribavirin at two schemes (co-treatment and post-treatment). Culture supernatants were harvested at 48 h post-inocubation and concentrations of cytokines were measured by ELISA. **(A)** Changes in TNF-α concentration. **(B)** Changes in IL-1β concentration. Data represent the mean ± SD of three experiments (*n* = 3); *p < 0.05, ***p < 0.002, ****p < 0.001. Uninfected cells were used as a mock control (Mock). PBS was used as a solvent control.

The influence of ribavirin on inflammation response was further evaluated in TBEV-infected A549 cells. At 48 h post-inocubation, the culture supernatants from the cells were collected for measurement of TNF-α and IL-1β release by ELISA. Figure 6A showed that TBEV infection led to strong induction of TNF-α. The peak levels of TNF-α were 108.52pg/mL (the co-treatment) and 68.81pg/mL (the post-treatment) in the culture supernatants from A549 cells infected with TBEV, respectively. Such TNF-α levels were significantly higher as compared with the uninfected cells (Mock; *p* < 0.001 for the co-treatment, *p* < 0.002 for the post-treatment). However, the influence of ribavirin on TBEV-mediated TNF-α release varied among the different treatment schemes. In response to the co-treatment of ribavirin, there was little or no reduction in TNF-α amounts. Whereas the post-treatment of ribavirin resulted in a substantial decrease in TNF-α amounts. Treatment with ribavirin at the dose of 20 or 50 μg/mL significantly reduced TNF-α amounts as compared with the untreated cells (0 μg/mL; *p* < 0.05). Conversely, there were no obvious inhibitory effects of ribavirin on IL-1β amounts in the culture supernatants from the infected A549 cells with the co-treatment as well as post-treatment of ribavirin (Figure 6B). PBS treatment showed no inhibitory effects on the TNF-α and IL-1β amounts in the culture supernatants from the infected cells. Ribavirin exerted differential inhibitory effects on the inflammatory cytokines and the post-treatment of ribavirin reduced TBEV-mediated TNF-α induction in A549 cells.

## Discussion

TBEV infection has raised public health concern due to the absence of effective antiviral treatments. With respect to its wide use as a therapeutic antiviral drug, whether ribavirin has anti-TBEV efficiency is taken into consideration. The current study demonstrated that ribavirin exhibited a potent antiviral action during TBEV infection *in vitro*, as evidenced by the inhibition of TBEV propagation as well as the initiation of antiviral and anti-inflammatory responses. Moreover, which stages of TBEV life cycle targeted by ribavirin as well as the molecular elements involved in the antiviral processes were analyzed and discussed.

The development of broad-spectrum antiviral compounds is in progress to improve treatment protocols against highly pathogenic arboviruses. Ribavirin is effective against the several viruses in the clinical management and a number of viruses *in vitro* with multiple effects on virus replication and propagation (Beaucourt and Vignuzzi, 2014). Previous studies showed that ribavirin was inhibitory to yellow fever virus, dengue virus, West Nile virus, Japanese encephalitis virus and TBEV in some cell cultures (Canonico et al., 1984; Huggins et al., 1984; Huggins, 1989; Neyts et al., 1996; Jordan et al., 2000; Crance et al., 2003). Here, we performed systematic experiments to investigate the safety and antiviral efficacy of ribavirin in the A549 and SH-SY5Y cells with TBEV infection. First, ribavirin displayed minor cytotoxicity on the cell lines and such characteristic enabled its clinical usage as an antiviral drug, which was beneficial for the evaluation of anti-TBEV action at a wide range of ribavirin doses in the different types of cells. Next, the dose-dependent inhibition of TBEV protein expression was observed in the infected A549 cells upon ribavirin treatment. In consistent with such inhibition, ribavirin indeed protected the infected cells by reducing the cytopathic effect. Then, the treatment of ribavirin significantly decreased TBEV titers in a dose-dependent manner in both A549 cells and SH-SY5Y cells. At the same time, the ribavirin treatment also exhibited the dose-dependent reduction of TBEV RNA levels. The markedly inhibitory effects of ribavirin on the viral RNA replication were observable in A549 and SH-SY5Y cells. These results demonstrated that ribavirin could strongly inhibit TBEV propagation in the susceptible cells at a non-cytotoxic dose range. In agreement with our results, on the basis of protein-drug interactions, ribavirin potently inhibited TBEV by targeting TBEV NS3 helicase (Singh and Somvanshi, 2009). Ribavirin significantly inhibited the proliferation of the highly virulent strain of the TBEV in the PK cell cultures (Krylova and Leonova, 2016). A recent study also reported that ribavirin exhibited inhibition toward highly and low-virulent strains of TBEV on SPEV cells (Leonova et al., 2020). However, 50 μM of ribavirin displayed no noticeable inhibitory effects on reducing TBEV titers in rat organotypic cerebellum slices (Lenz et al., 2018). We propose that culture models, virulent strains of TBEV and doses of ribavirin may be involved in the differences in anti-TBEV action of ribavirin.

The antiviral action of ribavirin on TBEV was further explored based on its influence in the viral life cycle. The time-of-addition assay was employed to determine which step(s) in the viral life cycle is blocked by an antiviral agent(s) (Chen et al., 2017; Aoki-Utsubo et al., 2018; Krylova et al., 2018). A549 and SH-SY5Y cells infected with TBEV were treated with ribavirin at the three schemes including co-treatment, post-treatment and pre-treatment. The TBEV titers, viral RNA levels and MxA mRNA levels were analyzed and compared. The TBEV titers were significantly reduced in the cells treated with ribavirin at the scheme of co-treatment as well as post-treatment. Conversely, no inhibitory effects on TBEV titers were found in the cells with ribavirin pre-treatment. In parallel with the reduction in TBEV titers, the viral RNA levels were markedly decreased in the cells treated with ribavirin at the scheme of co-treatment as well as post-treatment. These results suggest that ribavirin exhibits antiviral action by targeting TBEV replication stage (co-treatment and post-treatment) rather than early stage of virus penetration into cells (pre-treatment), implying that ribavirin may be used as a promising antiviral drug for the treatment of TBEV infection but not a preventive therapy.

The molecule events underlying the anti-TBEV action of ribavirin were preliminarily investigated in this study. Human MxA is an IFN-induced dynamin-like GTPase with broad antiviral activity through engagement of a diversity of viral proteins (Haller and Kochs, 2011; Mitchell et al., 2013). The expression of MxA in chronic hepatitis C played a role among the mechanisms underlying responsiveness to therapy of pegylated IFN in combination with ribavirin (Giannelli et al., 2004). Ribavirin was also reported to enhance IFN-α-induced mRNA and protein expression of MxA, which was a novel immune modulation mechanism during treatment of hepatitis C virus (Stevenson et al., 2011). It is thus interesting to investigate whether the antiviral action of ribavirin correlates with MxA. In consistent with the inhibitory effects of ribavirin on TBEV propagation, our results demonstrated that MxA mRNA levels were dose-dependently increased in TBEV-infected A549 cells with the co-treatment as well as the post-treatment of ribavirin, indicating that ribavirin may exhibit anti-TBEV action through induction of cellular antiviral response, in particular by up-regulating MxA expression. At the same time, STAT3 was also focused due to its distinct roles in regulating host immune responses and several viral diseases. It is known that STAT3 exhibits a proviral function in several viral infections, whereas STAT3 has an antiviral function in other viral infections (Chang et al., 2018). Moreover, STAT3 is proposed to be a potential target for antiviral therapy (Roca Suarez et al., 2018; Pandey et al., 2022). Inhibition of STAT3 signaling has been implicated in some cell lines during infection with several viruses including Marburg virus, human metapneumovirus, Ebola virus and Kaposi’s sarcoma-associated herpesvirus (Valmas et al., 2010; Mitzel et al., 2014; Harrison et al., 2021; Lee et al., 2023). These studies provide fundamental insights into the mechanisms of pathogenesis of viruses. Our results showed that TBEV potently inhibited the STAT3 phosphorylation in A549 cells, which may account for the viral survival and propagation and thereby establish infection. Recent studies have reported that STAT3 activation is responsible for the antiviral activities, such as STAT3 activation exerted an anti-enterovirus 71 activity; IL-22 suppressed infection of porcine enteric coronaviruses and rotavirus by activating STAT3 pathway; activation of STAT3 signaling was involved in inducing Sindbis virus E2 glycoprotein antibody-mediated viral suppression and viral clearance from neurons; antimicrobial peptide REG3G inhibited replication of Swine coronavirus-porcine epidemic diarrhea virus by up-regulating STAT3 pathway (Chang et al., 2017; Xue et al., 2017; Yeh et al., 2020; Fan et al., 2023). In accordance with the inhibition of TBEV production and the viral RNA replication by ribavirin, we observed that the ribavirin treatment distinctly enhanced the STAT3 phosphorylation in TBEV-infected A549 cells, implying that STAT3 activation might be a key event involved in the antiviral effect of ribavirin.

TBEV infection causes severe central nervous system diseases such as meningitis or encephalitis that is characterized by inflammation. Elucidation of cellular immune response to TBEV infection is important for understanding the viral pathogenesis and developing effective treatment of the diseases. The increased serum levels of TNF-α were found in patients of tick-borne encephalitis (Atrasheuskaya et al., 2003). Indeed, we found that TBEV infection led to strong induction of TNF-α in A549 cells. Ribavirin is known to possess immunomodulatory action. The roles of ribavirin in the induction of cytokines by some viruses were reported. For example, ribavirin suppressed activation of select inflammatory mediators triggered by Andes-virus (Khaiboullina et al., 2013). Treatment with ribavirin decreased the concentrations of TNF-α and IL-1β during murine hepatitis virus strain 3 infection *in vitro* and *in vivo* (Ning et al., 1998; Levy et al., 2006). Treatment of dengue virus-infected cells with ribavirin reduced TNF-α transcription (Rattanaburee et al., 2015). Ribavirin decreased the level of TNF-α and IL-1β in lung of mice infected with influenza virus (Liao et al., 2017). The influence of ribavirin in induction of pro-inflammatory cytokines TNF-α and IL-1β was thereby assessed in the TBEV-infected A549 cells. Our results showed that ribavirin significantly inhibited the production of TNF-α at the scheme of post-treatment, whereas it did not obviously diminish the production of IL-1β. Upon the post-treatment of ribavirin, the suppression of TNF-α release by TBEV-infected A549 cells was in parallel with the inhibition of TBEV propagation and viral RNA replication as well as the up-regulation of MxA mRNA expression and STAT3 phosphorylation, supporting that ribavirin may exhibit antiviral action by targeting TBEV replication stage. Our findings suggest that the suppression of TBEV-induced TNF-α release by ribavirin may attenuate inflammation *via* controlling pro-inflammatory cytokine-mediated immunoinflammatory events. Whether such suppression is associated with the inhibition of TBEV propagation by ribavirin deserves further study.

In conclusion, ribavirin may represent an effective drug toward TBEV infection for its potent inhibition of the virus propagation and limiting immune response in susceptible cell lines. Molecular bases of antiviral effect of ribavirin for TBEV need to be further investigated based on different virulent strains of TBEV and suitable animal models. Ribavirin’s precise antiviral mechanisms against TBEV remain to be elucidated and are implicated in the development of specific anti-TBEV treatments.

## Data availability statement

The original contributions presented in the study are included in the article/supplementary material, further inquiries can be directed to the corresponding authors.

## Author contributions

W-DT and H-LT conducted the study. H-RP analyzed the data. R-WR revised the manuscript. PZ supervised the study. L-JZ designed the study, wrote and revised the manuscript. All authors contributed to the article and approved the submitted version.

## Funding

This work was funded by the National Key Research and Development Program of China (2016YFC1202903) to L-JZ.

## Conflict of interest

The authors declare that the research was conducted in the absence of any commercial or financial relationships that could be construed as a potential conflict of interest.

## Publisher’s note

All claims expressed in this article are solely those of the authors and do not necessarily represent those of their affiliated organizations, or those of the publisher, the editors and the reviewers. Any product that may be evaluated in this article, or claim that may be made by its manufacturer, is not guaranteed or endorsed by the publisher.
